# Courtship choreography is stabilized among genetically isolated populations

**DOI:** 10.1093/beheco/arag048

**Published:** 2026-04-30

**Authors:** Nathan J Butterworth, Thomas E White, Blake M Dawson, Jesse Appleton, Clayton McDonald, Angela McGaughran, Gregory Markowsky, Keith M Bayless

**Affiliations:** School of Life and Environmental Sciences, Deakin University, 221 Burwood Hwy, Burwood, VIC 3125, Australia; Department of Ecological, Plant and Animal Sciences, La Trobe University, Plenty Rd, Bundoora, VIC 3086, Australia; School of Life and Environmental Sciences, University of Sydney, Camperdown, NSW 2050, Australia; Department of Entomology, Michigan State University, East Lansing, MI, United States; School of Earth, Atmospheric and Life Sciences, University of Wollongong, Northfields Ave, Wollongong, NSW 2500, Australia; School of Mathematics, Monash University, Wellington Rd, Clayton, VIC 3800, Australia; Te Aka Mātuatua, School of Science, University of Waikato, Private Bag 3105, Hamilton 3240, New Zealand; School of Mathematics, Monash University, Wellington Rd, Clayton, VIC 3800, Australia; Australian National Insect Collection, CSIRO National Research Collections Australia, Canberra, ACT 2601, Australia; ehduval@gmail.com

**Keywords:** sexual selection, courtship behavior, courtship evolution, behavioral diversification, diptera, geographic divergence, genetic isolation, genetic divergence

## Abstract

Sexual selection has sculpted diverse and intricate courtship displays throughout the animal kingdom, where failure to achieve the choreographic standards of a potential partner can be highly costly for reproductive success. Yet this raises a paradox: if there is strong selection for optimal display choreography within species, how do courtship displays begin to diverge? To address this, we measure how the choreography of courtship changes among three populations of the dancing dune fly—*Apotropina ornatipennis* Malloch (Diptera: Chloropidae)—a species in which males and females spend their days cavorting on Australia's hot sandy beaches. Merging population genetics with detailed quantification of the courtship display, we explore which elements of the display are the first to diverge between isolated populations, whether new behaviors arise rapidly, and whether sequence rearrangements occur in the modular structure of the display. We find that these tiny flies express courtship repertoires that rival some of the most sophisticated displays in the animal kingdom. Yet despite clear genetic and geographic isolation, the complex choreography of courtship is largely stable—with the frequency of “wing sweep” emerging as the only display element showing evidence of divergence among populations. In contrast to the notion that courtship behavior should be highly evolvable and rapidly diverge among allopatric populations, our findings suggest that complex courtship displays can remain remarkably stable over short evolutionary timescales.

## Teaser

Dancing dune flies perform dazzling courtship displays to attract mates. We studied whether these courtship displays vary between isolated populations. Despite genetic and geographic separation, display choreography remained remarkably stable. This suggests that complex courtship may resist rapid evolutionary change, helping us understand how courtship behaviors evolve—or stay the same—over time.

## Introduction

The animal kingdom abounds with an extraordinary variety of courtship displays that have evolved over millions of years to captivate the attention of receivers. Inadvertently, they have also captivated the attention of our own sensory systems (Darwin 1871; Bastock 1967; Thornhill and Alcock 1983; Eastwood et al. 1999; Shuker and Simmons 2014; Arnold et al. 2017; Cannon 2023). From the tandem runs of courting *Electrotermes* termites trapped in amber deposits 38 million years ago (Mizumoto et al. 2024), to the graceful pirouettes and bows of feral pigeons and their relatives (Columbiformes) (Roberts 1905; Boehm 1955; Fabricius and Jansson 1963; Goodwin 1966; Frith 1977), to the iridescent abdomen-shaking and leg-waving displays of over 100 described species of peacock spiders (Salticidae: *Maratus*) (Girard et al. 2015, 2021; Schubert 2020; Otto and Hill 2021). These intricate and high-stakes performances—where both males and females interact with the opposite sex through synchronized movements (see Gwynne and Simmons 1990; Amundsen 2000; Amundsen and Forsgren 2001; Kraaijeveld et al. 2007; Clutton-Brock 2009; Edward and Chapman 2011)—are crucial to reproductive success (Vinnedge and Verrell 1998; Shamble et al. 2009; White et al. 2020).

While the macro evolutionary patterns of courtship display evolution are increasingly well documented across the animal kingdom (eg, Spieth 1952; Goodwin 1966; Arnold 1972; Frith 1977; Kusmierski et al. 1997; Senter et al. 2014; Arnold et al. 2017; Ligon et al. 2018; Miles and Fuxjager 2018; Chen et al. 2019; Broder et al. 2021; Girard et al. 2021; Yukilevich 2021), we still have a rather limited understanding of the processes that generate this diversity over shorter timescales (West-Eberhard 1983; Ptacek 2000; Wiens 2000; Svensson and Gosden 2007; Wilkins et al. 2013; Mendelson et al. 2014; Arnold and Houck 2016; Mitoyen et al. 2019; Fuxjager et al. 2022; Schwark et al. 2022; MacGillavry 2025; Sibly and Curnrow 2025). Courtship displays are composed of multiple interacting components (Arnold et al. 2017), each potentially subject to stabilizing selection (conserving species-specific choreography) (Arnold et al. 2017), directional selection (driving diversification and elaboration), or fluctuating selection (which can likewise reinforce phenotypic stasis; Stroud et al. 2023). Yet the relative contributions of these selective regimes—and the timescales over which display elements originate, are lost, or are reordered—remain poorly resolved. Without this understanding, the evolutionary mechanisms behind even the most conspicuous divergences in courtship remain unexplained—such as why one species “pirouettes” (Fabricius and Jansson 1963) while its close congener “kangaroo hops” (Cramp 1958)?

Studying variation in courtship displays among natural populations can reveal how sexual selection, drift, and local adaptation shape signal evolution prior to reproductive isolation, offering a window into the earliest stages of behavioral divergence (Endler 1977; Foster and Endler 1999; Svensson and Gosden 2007; Mendelson et al. 2014; Iglesias et al. 2018; Gallagher et al. 2022). Empirical data shows ample evidence of divergence in courtship displays among populations (Luyten and Liley 1985; Kanmiya 1990; Paillette et al. 1997; Uy and Borgia 2000; Smith and Hunter 2005; Etges et al. 2006; Arbuthnott et al. 2010; Iglesias et al. 2018; Gallagher et al. 2022)—suggesting that they can diversify over short evolutionary timescales. This high evolvability of courtship traits has been reiterated by many experimental studies (West-Eberhard 1983; Gleason and Ritchie 1998; Mendelson and Shaw 2005; Snook et al. 2005; Zuk et al. 2006; Rogers and Greig 2008; Tinghitella 2008; Arbuthnott 2009; Ding et al. 2016; Han et al. 2016; Gallagher et al. 2022) and fits neatly into the general notion that behavioral phenotypes are particularly evolutionary labile (Gleason and Ritchie 1998; Blomberg et al. 2003; Arbuthnott 2009; Hernández et al. 2021). Yet, there are also many examples where courtship displays do not diverge substantially among populations but rather appear to be consistent and stabilized (Butlin et al. 1985; Gerhardt 1991; Noor et al. 2000; Watts et al. 2019; Moran et al. 2020). This aligns with macro evolutionary evidence of courtship stability—for example, the remarkable phylogenetic conservation of the bowing display in pigeons (Columbidae) (Goodwin 1966; Frith 1977), the straddle in *Lispe* flies (Muscidae) (White et al. 2020; Butterworth et al. 2021), and the tail-straddling walk of *Plethodon* salamanders (Plethodontidae) (Arnold et al. 2017). Altogether suggesting that courtship displays (or at least some components of them) can be subject to stasis or gradual evolution.

This raises the question: When and why should we expect to see divergence of courtship displays among contemporary populations? The extent and rate of among-population divergence for any given component of a courtship display will depend firstly on many extrinsic factors. For example, current and historic geographic variation in ecological characteristics (such as brightness, background motion, color, predation pressure, pathogens, resource availability and climate) will play a key role in shaping the extent of among-population diversification in courtship components by shifting male trait optima and female preference windows to align with the habitat (Fleishman 1988; Endler 1992; Butlin 1993; Day 2000; Yeh 2004). Specifically, as environments change across populations, the efficacy, costs, and information content of signals can shift, altering the optimal display through the eye of the receiver (ie, sensory drive sensu Endler 1992; but see also Peters et al. 2007; Chung et al. 2014; Heinen-Kay et al. 2015; Morier-Genoud and Kawecki 2015; Cummings and Endler 2018; Tinghitella et al. 2020; Wilson et al. 2021; Boughman and Servedio 2022). Similarly, geographic variation in selection on sensory systems for detecting resources, prey, or predators can fine tune visual preferences and sensitivity for cues that have not yet developed in the population and could facilitate the invasion of novel courtship behaviors.

Even in the absence of ecological differences among populations, courtship diversification can proceed depending on the form of selection on each component of the courtship display (ie, stabilizing, disruptive, fluctuating or directional) (Gerhardt 1991; Shaw and Herlihy 2000; Kirkpatrick and Nuismer 2004; Brooks et al. 2005; Oh and Shaw 2013; Selz et al. 2016; Arnold et al. 2017; Stroud et al. 2023) and the shape of the preference landscape (Butlin 1993; Mendelson et al. 2014). Importantly, the form of selection may also depend on the function of a given courtship component. Display components involved in species- or mate-recognition might be under strong stabilizing selection among populations, due to the potentially high costs of mating with the wrong species and hence exhibit stasis or diverge slowly (Butlin et al. 1985; McPeek et al. 2011; Wojcieszek and Simmons 2012). On the other hand, mate preference cues may be under disruptive or directional selection among populations, due to the costs of mating with locally maladapted individuals, and hence exhibit more evolutionary lability (Vortman et al. 2013; Selz et al. 2016; McClure et al. 2019). The possible rate of divergence will also be constrained by the network structure of the courtship display (Hebets et al. 2016)—in other words, the structural complexity of a courtship display will dictate its evolvability, and may even act as a stabilizing feature (Arnold et al. 2017). For example, behaviors that are connected and reinforce one another (ie, modular components) may evolve more slowly (Hebets et al. 2016; Arnold et al. 2017) and likewise with pluripotent behaviors that serve multiple functions such as the bowing display of pigeons which are used for both social aggression and sexual courtship in certain species (Frith 1977). Components that exhibit redundancy (similar cues with the same function) and degeneracy (different cues with the same function) may show the greatest evolutionary lability among populations (Hebets et al. 2016; Hoke et al. 2019), in line with the way that gene duplications promote neofunctionalization (Arnegard et al. 2010).

There is also a major role of genetics in the diversification of courtship displays (Arbuthnott 2009; Chenoweth et al. 2010; Cande et al. 2012; Cande et al. 2014; Yeh and True 2014; Ding et al. 2016; Rossi et al. 2020; Duckhorn et al. 2022). Complex polygenic traits are expected to evolve more slowly (Orr 2000) and evidence suggests that courtship traits are often polygenic (Cande et al. 2012; Yeh and True 2014; Rossi et al. 2020) *and* involve pleiotropic loci (Yeh and True 2014; Ding et al. 2016)—which may result in slow rates of divergence among populations that are hard to detect over contemporary timescales. In terms of population genetics, the extent of gene flow, drift, migration, and population size will also constrain how display traits diverge among populations (Endler 1977; Lande 1981; Thompson 1999; Sibly and Curnow 2025). With high migration and gene flow among large populations there may need to be very strong selection on courtship displays for populations to diverge in display traits (Endler 1973; Charlesworth et al. 1982; Kirkpatrick 1996; Thompson 1999). Despite this, there is evidence of such geographic divergence in displays occurring in nature even in panmictic populations (Iglesias et al. 2018). Nevertheless, studies that correlate patterns of population structure and gene flow with courtship diversification in natural populations remain rare (Wong et al. 2004; Svensson and Gosden 2007; Iglesias et al. 2018; Reda et al. 2025).

Overall, we have a good understanding of what *should* shape the remarkable diversification of courtship displays that we see at macroevolutionary scales. Yet we lack fundamental data from natural populations on how the entire courtship choreography diverges. Examples of novel courtship behavioral traits or rearrangements occurring among populations and over contemporary timescales are sparse relative to the diversity of courtship displays observed across the animal kingdom (Svensson and Gosden 2007; Svensson 2019; Gallagher et al. 2022; Gallagher et al. 2024), and few studies have considered the full scale of complexity in courtship displays (as per Lasbleiz et al. 2006; Scholes 2008a, 2008b; Arnold et al. 2017)—from qualitative differences in the presence/absence of behaviors, to quantitative changes in their frequencies and tempo, to changes in the sequence and arrangement of courtship modules. Also important to consider is that male courtship behavior does not occur in isolation—many male display elements are contingent on or modified by female behaviors, yet female behaviors are rarely included in courtship ethograms (as per Lasbleiz et al. 2006). Interpreting male displays in isolation risks mischaracterizing the structure of the interaction, particularly given that female responses are a primary selective force shaping male signal evolution (Ritchie 1996; Rodríguez et al. 2006; Thom and Dytham 2012). Importantly, female responses or preference windows may also diverge across populations even when male signals do not (Butlin 1993; Simmons et al. 2001; Mendelson et al. 2014; Tinghitella et al. 2020; Boughman and Servedio 2022; Yukilevich 2025), which should also be measured to ascertain whether the function of different male behaviors across populations remains consistent (Watts et al. 2019).

Here, we use a quantitative ethological framework to describe the courtship displays of the dancing dune fly *Apotropina ornatipennis* (Malloch 1923) (Diptera: Chloropidae), and to test how courtship divergence proceeds across natural populations. This species is commonly found along the beaches of the east coast of Australia. As habitats, Australia's beaches are particularly patchy, with a total of 10,000 beaches occupying 49.1% of the continent's coastline, interspersed by rocky outcrops, mangroves, and other habitats (Australia State of the Environment Report 2021). We expect that the patchy nature of these beach habitats restricts migration and gene flow among *A. ornatipennis* populations, and in turn that these allopatric populations will have rapidly evolved distinct differences in their courtship displays. By elucidating the precise mode of intraspecific diversification across the full suite of courtship components and choreography, we aim to provide insight into the mechanisms that drive the evolutionary diversification of courtship displays.

## Methods

### Animals

**Figure 1:**
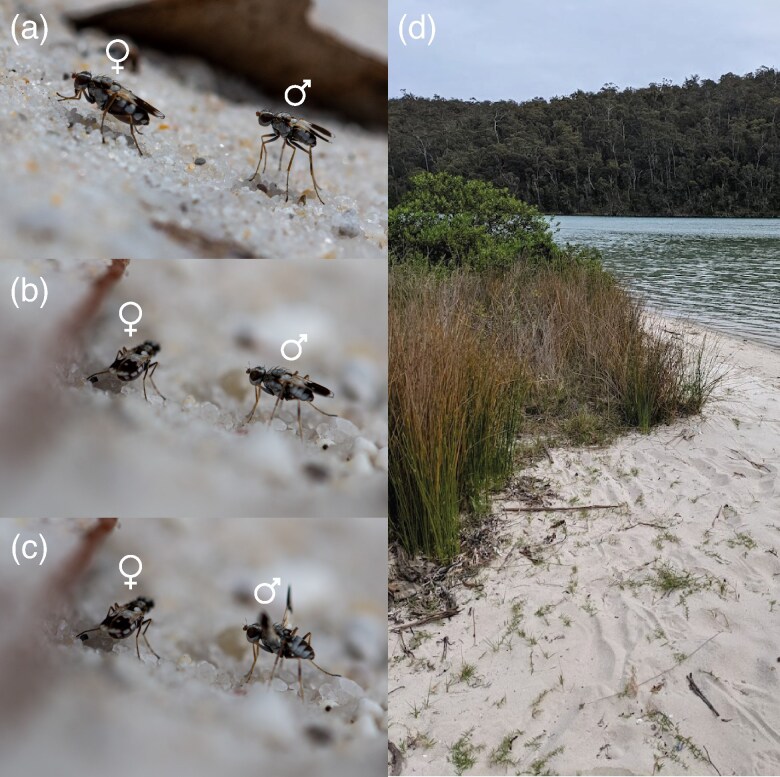
The dancing dune-fly, *Apotropina ornatipennis* (Diptera: Chloropidae). a) Orienting, b) Face-off, c) Wing-sweep, d) typical habitat.

*Apotropina ornatipennis* Malloch (1923) (Diptera: Chloropidae) is one of twenty-two described *Apotropina* from Australia (Ang et al. 2023). The species inhabits beaches on the eastern coast of Australia (it can be found between Sydney and Eden in New South Wales but its full range remains unknown) and is most abundant in sandy areas of the beach with coastal grasses (Poales). The larval life-history is entirely unknown, and the present authors have never observed any female oviposition. Adults are active year-round though at greatly reduced population densities in the winter (estimates of 1 to 2 individuals/m^2^) (personal observations). Population size peaks in the warmer spring and summer months, with aggregations ranging from estimates of 1 to 30 individuals/m^2^ (personal observations). Females spend their days foraging and often stand in the shade under fallen debris and leaves, while males actively run across the sand and vigorously court any females they encounter (Fig. 1; Supplementary Video). Females are usually larger than males, but both sexes exhibit pigmented wings, with patterns that are superficially sexually monomorphic (see Figure S3) and are vibrated frequently by both sexes during courtship. The males will also engage in male-male combat, which appears to involve direct contact between the proboscis of both males (see Supplementary Video). *A. ornatipennis* is often the dominant species where it occurs—alongside terrestrial amphipods (Talitridae) and various small ants. Predators include spiders and flies of the genus *Lispe* (Diptera: Muscidae) which have been observed to opportunistically kill roaming *A. ornatipennis* males (personal observations). Most commonly, individuals occur in conspecific masses, with few opportunities for males to encounter other dipteran species and to misdirect courtship.

### Population genetics

To ascertain the genetic structure of populations among beaches, flies were collected between 24th of October and 17th of December 2021 from seven sites (Table 1), euthanized with ethyl acetate vapour within 8 h of capture and placed into 2.5 mL plastic tubes containing 90% ethanol and stored at −4 °C in the laboratory for up to eight weeks. A subset of specimens was taxonomically identified by comparison to the holotype and paratypes in the Australian National Insect Collection (Canberra, Australia).

**Table 1:** Summary of collection localities and population genetic parameters.

Site	Code	Latitude	Longitude	Date collected	*N*	H_O_	H_E_	F_IS_	F_ST_	SNP_PRIV_
**Greenpatch**	GR	−35.1365	150.72346	17/12/2021	23	0.139	0.19	0.298	0.0000	2,316
**Wairo**	WA	−35.4276	150.41645	24/10/2021	9	0.088	0.11	0.161	0.3547	122
**Haywards**	HA	−36.4053	150.06543	24/10/2021	12	0.109	0.125	0.106	0.2628	11
**Beares**	BE	−36.4324	150.07836	24/10/2021	11	0.106	0.125	0.135	0.2619	16
**Merimbula**	ME	−36.9033	149.91	23/10/2021	19	0.115	0.131	0.126	0.2236	42
**Middle**	MI	−36.8915	149.92931	12/12/2021	10	0.111	0.133	0.143	0.2163	8
**Severs**	SE	−36.9526	149.90857	07/12/2021	10	0.108	0.13	0.158	0.2320	14

Each individual fly (9 to 23 per population) was placed in a single well of a 96-well plate with 70% ethanol, and sent to Diversity Arrays Pty Ltd (Canberra, Australia) for a high-density DArTSeq assay (∼ 2.5 million sequences/sample used in marker calling). The DArTSeq extraction and sequencing methods are detailed in Kilian et al. (2012) and Georges et al. (2018).

To ensure appropriate DNA fragments were used for subsequent sequencing, restriction enzyme digestion was optimized for *A. ornatipennis* using multiple restriction enzyme combinations and eight specimen replicates. Following sequencing of these test specimens, the optimal restriction enzyme pair was identified as PstI-HpaII, based on the fraction of the genome represented, while controlling for average read depth and the number of polymorphic loci. This restriction enzyme combination was used for all subsequent digestions. Following digestion, all sequence fragment libraries were ligated with Illumina sequencing adaptors and sequenced on an Illumina HiSeq2000 platform.

Short-read sequences were processed using the DarTseq bioinformatic pipeline (Georges et al. 2018), which performs filtering and variant calling, to generate final genotypes. While some parts of the sequencing and analysis protocol are proprietary and cannot be provided, the use of the DArTSeq platform for studies of genetic diversity and structure is widespread in the field (Popa-Báez et al. 2020; Hoffman et al. 2021; Jaya et al. 2022; Butterworth et al. 2023) and is reproducible.

### Genetic analysis

The DArTseq dataset contained a total of 38,905 SNPs across 94 individual flies (Supplementary Material S1). The data were then filtered with the “dartR” package version 1.9.9.1 (Gruber et al. 2018) in R version 4.5.0 (R Core Team 2025). We filtered the DArTseq dataset by reproducibility (proportion of technical replicate assay pairs for which the marker score was consistent) at a threshold of 0.98, then by call rate (proportion of samples for which the genotype call was not missing) at a threshold of 0.95, and finally by minor allele count at a threshold of 0.02 (MAC less than the threshold are removed). This resulted in a filtered dataset of 94 individuals, 7,856 SNPs, and 1.19% missing data.

We used R for all analyses of genetic diversity. We applied the “basic.stats” function of the “hierfstat” package version 0.5 to 10 (Goudet et al. 2005) to calculate average observed heterozygosity (HO), expected heterozygosity (HE), and inbreeding coefficients (FIS). We also used the “betas” function from “hierfstat” to calculate population-specific FST values (Weir and Goudet 2017).

Using R, we assessed population structure by AMOVA using the function “poppr.amova” with the “ade4’ implementation from the “poppr” package version 2.9.3 (Kamvar et al. 2014). To test whether populations were significantly different, we used a randomization test on the AMOVA output with 1,000 permutations (Excoffier et al. 1992) using the function “randtest” from the package “ade4’ version 1.7 to 18 (Thioulouse et al. 2018). We then conducted pairwise comparisons of FST values between populations using the “gl.fst.pop” function from the “dartR” package with 10,000 bootstrap replicates.

Genetic distances between individuals were examined using Nei's distances, and a dendrogram with 1,000 bootstrap replicates was created with the “aboot” function of the “poppr” package, and the “ggtree” function of the package “ggtree” (Yu 2020). We then used the “glPca” function from the “adegenet” package version 2.1.5 (Jombart 2008) to determine whether genetic differences between individuals (as represented by principal components) were structured according to their populations.

To test for isolation by distance, we performed a Mantel test using the function “gl.ibd” from the “dartR” package in R. This compared linearized genetic distances between populations (calculated using “StAMPP” version 1.6.3; Pembleton et al. 2013) against Euclidean geographical distances (calculated using “vegan” version 2.5 to 7; Oksanen et al. 2013).

To calculate individual admixture coefficients, the filtered SNP data were converted into the STRUCTURE format (“.str”) using the “gl2faststructure” function from the “dartR” package, then into the “.geno” format using the “struct2geno” function of the “LEA” package version 3.1.4 (Frichot and Francois 2015). We then ran sparse nonnegative matrix factorization on these data with the “sNMF” function from “LEA” to examine genetic clusters in the data. We analysed K values (ie, cluster numbers) of 1 to 10, with 100 replications for each K value, and used the cross-entropy criterion to determine the value of K that best explained the results.

### Quantifying courtship

From the seven sites used for genetic analysis, three were chosen for detailed analysis of courtship patterns. Two adjacent populations (Severs Beach and Middle Beach) that likely exchange migrants and should therefore have relatively higher gene flow, were compared with the most geographically distant population (Greenpatch Beach), where the proportion of migrants and gene flow would be expected to be lowest (see Supplementary material; Table S1). With this comparison, we would expect the Severs and Middle beach populations to exhibit most similarity in courtship displays and be highly distinct from the courtship displays observed in the Greenpatch populations.

At all beaches, filming occurred under clear conditions between the daylight hours of 0900 and 1500 and between the 1st and 17th of December 2021. Behavior was filmed under natural light and temperature conditions with a Google Pixel 5 (12.2 MP) or iPhone 11 (12 MP) camera recording at 60 frames per second. Notably, because courtship was filmed by observers who were not concealed, the presence of a large mammal observing the flies may have led to behavioral biases in courtship displays. Filming began when a male approached a female and continued until one or both flies left the area and could no longer be observed. Once video footage was obtained, slow-motion playback with Adobe Premiere Pro allowed us to describe all inter- and intra-sexual interactions (Table 2). For each male-female pair (*n* = 15 per population) we used Solomon Coder 17.03.22 (Péter 2017) to score the durations and frequencies of all male and female behaviors during interactions. All scoring was done by a single researcher (JA). Across both males and females, we recorded 18 behaviors that occurred during the courtship display. These behaviors were not all mutually exclusive as different body parts could be used simultaneously (Table 2)—for example, males could “orient” and “wing-vibrate” at the same time.

**Table 2:** Ethogram of courtship behaviors of *Apotropina ornatipennis*.

Behavior	Description	Figure
**Chasing (M)**	The male engages in an ambulatory pursuit of the female as she moves away from him.	…
**Face off (M)**	The male stands motionless facing directly toward the female and within her visual field, with <3 cm distance between them.	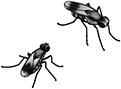
**Foraging (M/F)**	The male or female extends their proboscis to contact the substrate during the courtship interaction.	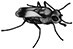
**Foreleg touch (M)**	The male contacts the body of the female with his foreleg.	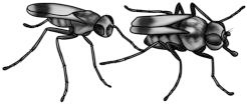
**Kick (F)**	The female kicks the male with one of her mid- or hind-legs.	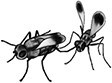
**Orient (M)**	While the female is stationary the male strafes around her, often performed in conjunction with a wing flash.	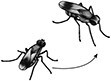
**Preening (M/F)**	The male or female preens themselves during the courtship interaction, this often involves rubbing the legs together and over the head.	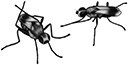
**Proboscis touch (M)**	The male contacts the female with his proboscis.	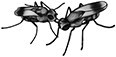
**Single wing (M/F)**	The male or female quickly and repeatedly extends and retracts a single wing (a ∼45-degree angle).	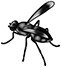
**Standing (F)**	The female stands motionless while in the vicinity of a male. Sometimes performed in conjunction with wing flapping.	…
**Straddle (M)**	The male holds onto the abdomen and/or wings of the female with both his forelegs.	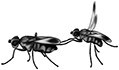
**Turn (F)**	While stationary and being courted by a male, the female turns her body in any direction.	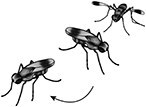
**Walking (F)**	The female disengages and walks away from the male.	…
**Wing flap (M/F)**	A constant flapping of both wings via rapid extension to 45-degrees and retraction. Is exhibited by both sexes. Individuals will wing flap even in the absence of other conspecifics, and always at a much slower speed than wing vibrating.	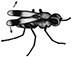
**Wing flash (M)**	The male vertically rotates both wings so that the black pigmented region is roughly at a 45-degree angle to the ground. Simultaneously the male horizontally extends both wings perpendicular to his body. This has the effect of displaying the full black pigmented region directly towards the female. Often performed in conjunction with orienting.	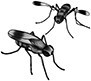
**Wing sweep (M)**	The male extends his wings rapidly so that they are perpendicular to his body. Simultaneously he rotates the wings vertically so that the black pigment is facing directly toward the female. The male then slowly (relative to the speed of other behaviors) vertically moves the wings 90-degrees until they are at a straight angle from his head. He then brings them down rapidly to their original position and repeats the behavior several times.	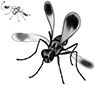
**Wing touch (M)**	The male brings his wings forward so that the posterior edge reaches beyond his head, and rapidly and repeatedly vibrates both wings while seemingly contacting the female's body (usually her wings or abdomen).	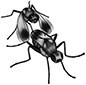
**Wing vibrate (M)**	The male rapidly extends and retracts his wings repeatedly in the horizontal plane to produce a vibrating effect.	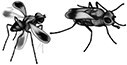

Behaviors by male (M), female (F), or both sexes (M/F).

Over two weeks (and more than 100 total hours) of filming flies, we were only able to observe a single pair proceed all the way to copulation. This means that 44/45 of the measured courtship interactions were from unsuccessful male-female pairs. Nevertheless, male-female interactions all involved prolonged back and forth assessment between the sexes before one partner or the other left (the median interaction time was 92 s, and the average interaction time was 148 s)—suggesting mutual assessment and female engagement in the courtship display. All recorded courtship components have evolved under selection in these natural populations and although we may devise clearer results (particularly for female preference) if we could also assess how successful males differed in courtship among populations, this was not possible to achieve as we did not observe enough copulations within the projects timeframe. Crucially, because we observed one complete courtship sequence through to mating, we can at least confirm that the behaviors recorded represent the majority of the display associated with successful mating. These observations also confirm that mating does occur on the beach, in the same habitat where courtship interactions take place. Therefore, we are confident that the interactions analysed accurately represent the natural courtship display and behavioral sequence of *A. ornatipennis*.

### Courtship statistical analysis

To determine whether the frequency with which behaviors occur within populations varied among populations, we qualitatively compared the proportion of flies exhibiting display behaviors among populations (number of flies that displayed behavior/total number of flies assayed in each population) (all observed behaviors; *N* = 18; Table 2).

To assess quantitative differences in the temporal patterns of male courtship displays among populations, we analysed individual male behaviors (ie, orient and wing-sweep in isolation, as opposed to the combined “orient-wing-sweep”; Table 2) and only those that occurred above 50% frequency within a population (*N* = 7), which allowed replication to be high enough for quantitative analysis. Although face-off and chasing met this threshold, they were excluded as they do not represent active courtship signals—face-off reflects a precondition for courtship rather than an active display element, while chasing is a locomotor state driven by female movement rather than male signaling investment. For each male-female courting pair we measured the following aspects of wing flash, wing sweep, wing vibrate, wing flap, and orient: (i) the mean bout duration as a proportion of the total interaction time (mean behavior bout duration/total interaction duration), (ii) the frequency (number of times a behavior occurred/total number of all behavioral occurrences), and (iii) the mean inter-bout interval as a proportion of the total interaction time (mean duration of time between behavior bouts/total interaction duration). All data were continuous proportions, so we used beta regression (betareg package in R; v. 3.2 to 4, Cribari-Neto and Zeileis 2010) to assess how each of these metrics varied among populations followed by “ANOVA” type III from the “car” package (v. 3.1-1, Fox and Weisberg 2018). Behavioral measures were expressed as proportions of total interaction time to account for natural variation in interaction duration among pairs—since males had variable access to female attention among interactions, the proportion of time invested in a behavior reflects courtship effort more meaningfully than absolute duration.

To assess whether female responses to male behaviors varied among populations, we compared the frequency of male wing-sweep and wing-flash against mean proportional duration per female walking bout (mean proportion of walking time per bout/total interaction time) as a sign of interest. This was informed by the stationary behavior of the single mated pair we observed where reduction in female movement appeared key to mating success—the female remained stationary for 92% of the 140 s interaction time. We therefore consider the lack of female movement as a receptivity response as per previous studies of fly courtship (White et al. 2020). We also compared the mean bout duration of male wing vibration against the mean bout duration of female wing flapping which is a common female response to courtship in flies (eg, Butterworth et al. 2019). Because data were all in the form of continuous proportions, we used beta regression followed by “ANOVA” type III, as described above.

Lastly, we assessed whether there was divergence in the structure of the courtship sequence among populations, and whether there was a relationship between genetic distance and divergence in the overall courtship sequences. Following Green and Patek (2018) we first used the igraph network analysis package (v.2.1.4, Csárdi and Nepusz 2006) to summarize behavioral sequence data into adjacency matrices for each population, where each row and column in the matrix corresponded to one of 41 behavioral combinations (41 × 41 matrix). Each cell in this matrix corresponded to the number of times, across the dataset, that one behavior from an individual transitioned to a subsequent behavior from that individual. We identified transitions that were more frequent than expected by chance (ie, the nonrandom components of the display) using permutation procedures for sequential behavioral analysis (see Bakeman et al. 1996; Green and Patek 2018). This enabled us to isolate only the significant transitions. From these nonrandom transition matrices, we then used Kullback-Leibler divergences (commonly employed for comparing Markov matrices in information theory; Rached et al. 2004) to compare distances in courtship display structure between individuals within- and among-populations. We used noninformative priors for the Dirichlet distribution using “KL.Dirichlet()” from the R package “entropy” (v.1.3.2, Hausser et al. 2012) to calculate divergence between vectorized Markov courtship transition matrices between pairs of individuals (ie, comparing the courtship structure of Greenpatch 1 to Greenpatch 2, then Greenpatch 1 to Severs 1, etc). To ascertain whether courtship displays divergence is related to genetic distance we then correlated these mean inter-individual courtship distances (as calculated by Kullback-Leibler divergences of transition counts per individual courting pair) with the mean inter-individual genetic distances (as calculated by Kosman distances; Kosman and Leonard 2005).

## Results

### Populations are genetically isolated and exhibit low levels of gene flow

**Figure 2:**
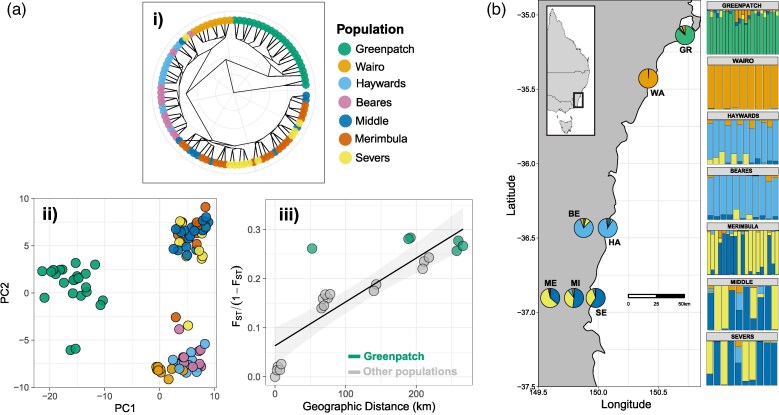
Population genetic metrics among several populations of *A. ornatipennis* (Diptera: Chloropidae). a) Genetic diversity as represented by (i) Nei's genetic distances, (ii) Principal component analysis, and (iii) A mantel test of genetic isolation by geographic distance. b) The mean admixture proportions of populations of *Apotropina ornatipennis* (Diptera: Chloropidae) that were sampled in the present study. The admixture pie charts plotted on the map represent population averages. The bar plots presented on the right reflect individual admixture proportions, sorted by population, where each bar represents a single individual. Full population names are provided in Table 1.

There was clear genetic differentiation among populations based on Nei's genetic distances (Fig. 2.a.i) and principal component analysis where the first two components explained 26.8% of the total variation with clear separation of populations (Fig. 2.a.ii). Results of the AMOVA further indicated that populations were differentiated with 14% of genotypic variation coming from among-individual differences within populations, and 21% from among-population differences (FST = 0.2043, *P* = 0.001). Pairwise F_ST_ values ranged from 0.014 to 0.280 (all *P*-values < 0.01) and were consistently highest for comparisons involving Greenpatch populations (Supplementary material; Table S1). The Mantel test indicated statistically significant correlation between genetic differentiation and geographic distance between sampled populations (*P* < 0.01) (Fig. 2.a.iii)—though Greenpatch contrasted this overall pattern, as it was distinct even from its closest population (Wairo Beach—within 43 km) and also had a high number of private alleles (Table 1). The sNMF analysis identified five genetic populations as the most likely, and Greenpatch and Wairo showed largely discrete clusters with limited admixture for *K* = 5 (Fig. 2b). In contrast, the remaining populations showed more mixing, with shared ancestry highest at local scales (ie, between Beares and Hayward Beach and between Merimbula, Middle, and Severs Beach).

### The precopulatory courtship display is highly complex

Courtship was highly complex consisting of 18 discrete behaviors (Table 2), and 41 behavioral states (Table 6).

### Courtship choreography is stable among genetically isolated populations

**Figure 3:**
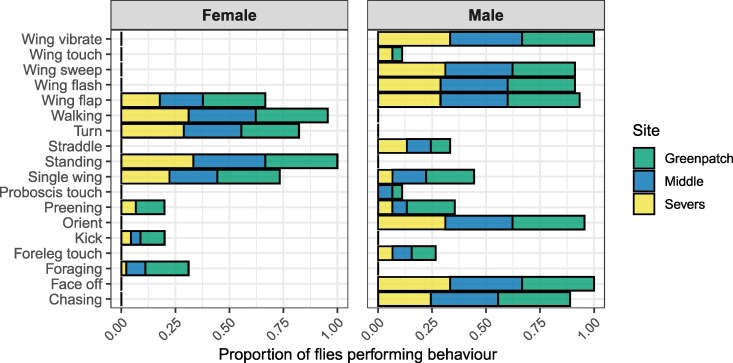
The proportion of flies (out of all 45 males and females) exhibiting the 18 observed behaviors expressed during courtship. The relative contributions from each beach to the total proportions are represented in color. The left panel shows females and the right panel shows males.

We found no evidence of divergence among the three tested populations regarding the innovation of new behaviors, or changes in the proportion of males performing any display components. All 18 display behaviors were observed in each population at similar proportions (Fig. 3). Active male courtship behaviors that occurred in more than 50% of pairs (across all sites) were used for downstream quantitative analysis among populations (wing flash, wing sweep, wing flap, wing vibrate, orient, walking).

**Figure 4:**
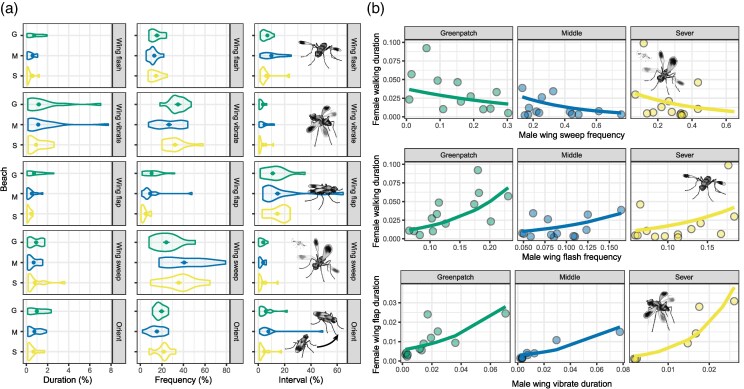
a) Predicted means and standard errors of the bout duration, frequency, and inter-bout interval of key male display components from the beta regression models (Table 3). b) Predicted fits of the beta regression models (Table 5) to the correlations between male display behaviors (wing sweep, wing flash, and wing vibrate) and female responses (walking and wing flap).

We found evidence that wing-sweep frequency differed among populations (Table 3; Fig. 4a). This was due to a lower frequency of wing-sweep at Greenpatch than the other two populations and was driven by 3/15 individuals at Greenpatch Beach that only performed a single wing sweep during the display (Greenpatch 10, 12, and 14). However, wing sweep made up 50% of all behaviors expressed by Greenpatch 7—so not all males at Greenpatch had low wing sweep frequencies. There were no significant differences in any other temporal patterns of the other main display components, including their duration, frequency, or interval (Table 3; Fig. 4a), suggesting largely consistent temporal patterning among populations. Notably however, we identified high levels of variation in temporal patterning within- and among-populations.

**Table 3:** Results of type III analyses of variance testing the effects of population (3 levels) and behavior (5 levels) on three behavioral metrics: mean proportional bout duration, behavioral frequency, and inter-bout interval (Fig. 4b).

	Duration			Frequency			Interval		
Full model	*χ* ^2^	df	*P*	*χ* ^2^	df	*P*	*χ* ^2^	df	*P*
Population	2.44	2	0.295	1.69	2	0.429	2.69	2	0.261
Behavior	7.59	4	0.108	87.24	4	**<0**.**001**	28.16	4	**<0**.**001**
Population × Behavior	1.52	8	0.992	49.39	8	**<0**.**001**	6.56	8	0.584
**Wing flash**									
Population	4.01	2	0.134	4.22	2	0.121	6.13	2	0.047
**Wing vibrate**									
Population	0.97	2	0.616	7.15	2	0.028	3.2	2	0.202
**Wing flap**									
Population	4.51	2	0.105	7.59	2	0.023	1.11	2	0.573
**Wing sweep**									
Population	1.74	2	0.419	13.14	2	**0**.**001**	1.22	2	0.543
**Orient**									
Population	3.44	2	0.179	3.24	2	0.198	7.24	2	0.0268

For each male–female courting pair we calculated: (1) mean bout duration, expressed as the proportion of total interaction time (mean behaviour bout duration/total interaction duration); (2) frequency, expressed as the proportion of behavioral occurrences (number of occurrences of a behavior/total number of behavioral events); and (3) inter-bout interval, defined as the mean time between successive bouts of a given behavior, expressed as a proportion of total interaction time (mean time between bouts/total interaction duration). The full model tested the effects of population, behavior, and their interaction. Additional rows show separate models for each behavior testing the effect of population. Bold values indicate statistically significant results after Bonferroni correction (*α* = 0.01).

We found clear relationships between female preference proxies and male behaviors (Tables 4 and 5, Fig. 4b). Wing sweep frequency and female walking bouts were negatively correlated (either females elicit male wing sweep when they stand, or male wing-sweep enhances female interest). Wing flash frequency and female walking bouts were positively correlated (either males use wing flashing to grab the attention of walking females, or male wing flash is a key component that causes female walking). Male wing vibrate bout duration and female wing flap bout duration were positively correlated (either male wing vibration elicits longer female wing flap bouts, or vice versa). Interestingly, in the one interaction we observed to proceed to mating (Severs 1), the male spent the greatest proportion of his time (34%) in wing sweep, likewise the male in one of the longest interactions before female rejection (576 s; Greenpatch 7) spent 50% of his time on wing sweep—thus, wing sweep may be an important reflection of male courtship effort and subsequent female engagement in the display.

**Table 4:** Results of the analysis of variance (type III) for the effects of male display traits (wing sweep frequency, wing flash frequency, and proportion time spent wing vibrating) on female responses (proportion time spent walking and proportion time spent wing flapping) (Fig. 4b).

Model, parameter	ANOVA (Type iII)		
Female walking proportion ∼ Male wing sweep frequency × Population	*χ* ^2^	df	*P*
Male wing sweep frequency	8.59	1	**0**.**003**
Population	11.46	2	**0**.**003**
Male wing sweep frequency × Population	5.54	2	0.063

Female walking proportion ∼ Male wing flash frequency × Population	*χ* ^2^	df	*P*
Male wing flash frequency	20.15	1	**<0**.**001**
Population	0.18	2	0.912
Male wing flash frequency × Population	2.65	2	0.266

Female wing flap proportion ∼ Male wing vibrate proportion × Population	*χ* ^2^	df	*P*
Male wing vibrate proportion	23.51	1	**<0**.**001**
Population	6.54	2	**0**.**038**
Male wing vibrate proportion × Population	24.27	2	**<0**.**001**

Bold numbers indicate significant values.

**Table 5:** Results of separate beta regression models estimating the effect of male display traits (wing sweep frequency, wing flash frequency, and proportion time spent wing vibrating) on female responses (proportion time spent walking and proportion time spent wing flapping) (Fig. 4b).

Model, parameter	Estimate	SE	*Z*	*P*	*R* ^2^
**Female walking proportion ∼ Male wing sweep frequency × Population**					
Intercept	−2.35	0.39	−5.98	**<0**.**001**	0.33
Male wing sweep frequency	−6.84	2.33	−2.93	**0**.**003**	…
Middle Beach	−1.77	0.58	−3.06	**0**.**002**	…
Severs Beach	−1.77	0.69	−2.54	**0**.**011**	…
Male wing sweep frequency × Middle Beach	5.96	2.59	2.29	**0**.**022**	…
Male wing sweep frequency × Severs Beach	5.92	3.03	1.96	0.051	…

Female walking proportion ∼ Male wing flash frequency × Population					
Intercept	−5.41	0.52	−10.44	**<0**.**001**	0.41
Male wing flash frequency	15.76	3.51	4.49	**<0**.**001**	…
Middle Beach	0.03	0.76	0.04	0.971	…
Severs Beach	0.31	0.78	0.4	0.689	…
Male wing flash frequency × Middle Beach	−4.68	6.39	−0.73	0.464	…
Male wing flash frequency × Severs Beach	−9.65	5.99	−1.61	0.107	…

Female wing flap proportion ∼ Male wing vibrate proportion × Population					
Intercept	−5.26	0.19	−26.6	**<0**.**001**	0.82
Male wing vibrate duration	23.63	4.87	4.85	**<0**.**001**	…
Middle Beach	−0.57	0.31	−1.89	0.071	…
Severs Beach	−0.93	0.41	−2.27	**0**.**024**	…
Male wing vibrate duration × Middle Beach	−0.4	7.22	−0.06	0.955	…
Male wing vibrate duration × Severs Beach	89.89	18.59	4.83	**<0**.**001**	…

Bold numbers represent statistical significance (*P* < 0.5).

However, while the correlation between female responses and male behavior was broadly consistent among populations, the strength of the relationship differed. There was a marginally significant interaction between male wing sweep frequency and population for female walking proportion (Table 4), whereby relationships were slightly more negative at Middle Beach and Severs Beach than at Greenpatch Beach (Table 5; Fig. 4b). In addition, the interaction between male wing vibrate proportion and population for female wing flap proportion was strongly significant (Table 4), driven specifically by a markedly stronger positive relationship in Severs Beach compared with Greenpatch, while Middle Beach—which is geographically proximate to Severs Beach—did not differ significantly from Greenpatch (Table 5; Fig. 4b). Together, these results suggest that there is some potential variation in the strength of female responses among populations—though in a manner that is not consistent with the strong patterns of genetic isolation.

**Figure 5:**
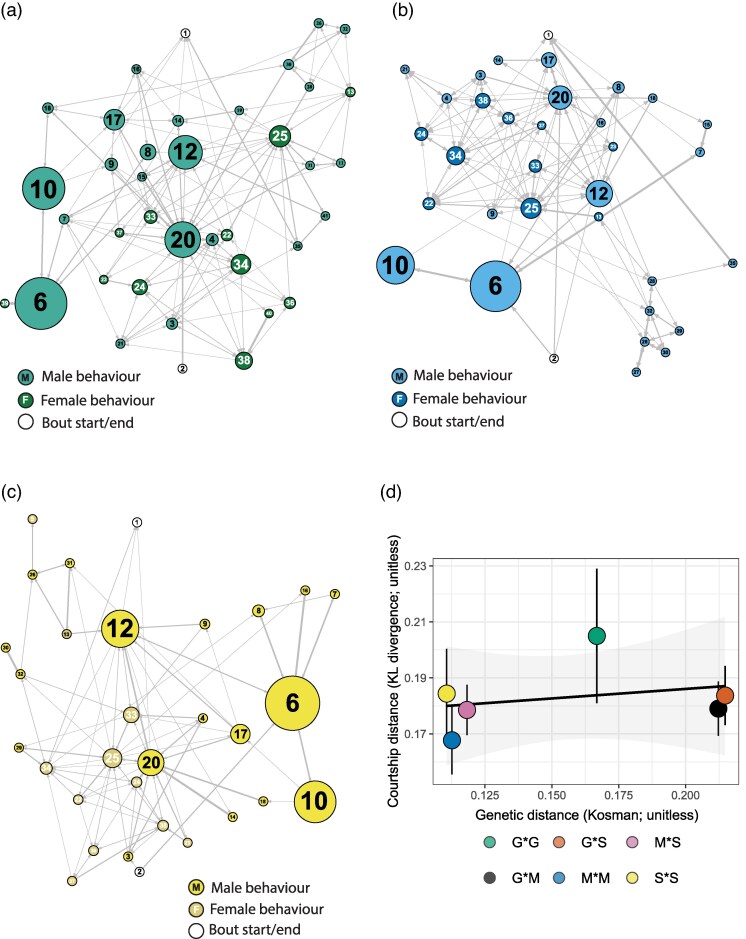
Sequential analysis of courting male-female pairs at a) Greenpatch Beach (G), b) Middle Beach (M), and c) Severs Beach (S). Circles with numbers represent discrete male and female behaviors (see Table 6). Circle size is scaled to the degree centrality of the behavior (percentage of total courtship behaviors), lines represent significant transitions between behaviors, and line width is scaled to transitional probability (0 to 100%). Color dictates whether the behavior was performed by males or females; see legend of each plot. d) Mean inter-individual courtship distances ± SE (as calculated by Kullback-Leibler divergences of transition counts, 1 as pseudocount) against mean inter-individual genetic distances (individual genetic distances calculated by Kosman distance; Kosman and Leonard 2005) of each population pair. No clear relationship was observed between genetic distance and courtship distance (*R*^2^ = 0.074).

Finally, we found no evidence of divergence in the choreography or sequence of courtship displays among populations (Fig. 5). Based on Markov analysis, the central well-connected nodes (behaviors) were the same across populations—with transitions occurring most frequently between the behaviors Face-off (6), Face-off-Wing-sweep (10), Orient-wing-vibrate (20), Face-off-Wing-vibrate (12), Orient-Wing-flash (17), Walking (34), and Wing-flap-Walking (38) (Table 6). Furthermore, no clear distinction in sequence across populations was detected based on Kullback-Leibler divergences, which were similar irrespective of the geographic distance of populations (mean KL divergences: Greenpatch × Middle: 0.179, Greenpatch × Severs: 0.184, Middle × Severs: 0.179) and we found no clear correlation between genetic distance and courtship distances (*R*^2^ = 0.074; Fig. 5d).

**Table 6:** List of behavioral states (defined as sustained combinations of simultaneously expressed behaviors) in the sequential analysis (Fig. 5a–c).

Number	Behavior
1	Bout-end
2	Bout-start
3	Chasing-(M)
4	Chasing-Wing-flap-(M)
5	Copulation-(F)
6	Face-off-(M)
7	Face-off-Single-wing-(M)
8	Face-off-Wing-flap-(M)
9	Face-off-Wing-flash-(M)
10	Face-off-Wing-sweep-(M)
11	Face-off-Wing-touch-(M)
12	Face-off-Wing-vibrate-(M)
13	Kick-(F)
14	Orient-(M)
15	Orient-Single-wing-(M)
16	Orient-Wing-flap-(M)
17	Orient-Wing-flash-(M)
18	Orient-Wing-sweep-(M)
19	Orient-Wing-touch-(M)
20	Orient-Wing-vibrate-(M)
21	Single-wing-(M)
22	Single-wing-Standing-(F)
23	Single-wing-Turn-(F)
24	Single-wing-Walking-(F)
25	Standing-(F)
26	Straddle-(M)
27	Straddle-Single-wing-(M)
28	Straddle-Wing-flap-(M)
29	Straddle-Wing-flash-(M)
30	Straddle-Wing-sweep-(M)
31	Straddle-Wing-touch-(M)
32	Straddle-Wing-vibrate-(M)
33	Turn-(F)
34	Walking-(F)
35	Wing-flap-(M)
36	Wing-flap-Standing-(F)
37	Wing-flap-Turn-(F)
38	Wing-flap-Walking-(F)
39	Wing-swing-Standing-(F)
40	Wing-swing-Walking-(F)
41	Wing-vibrate-(M)

## Discussion

There are almost as many unique courtship displays as there are animal species (Bastock 1967; Frith 1977; Arnold et al. 2017; Cannon 2023) yet we still know little about how such diversity arises in the face of constraining forces such as stabilizing selection, pleiotropy, and structural interactions between courtship elements. Here, we investigated courtship divergence among three populations of the dancing dune fly *Apotropina ornatipennis* (Diptera: Chloropidae). Contrary to our expectation that courtship behavior should diverge rapidly among allopatric populations, most measured aspects of courtship were consistent among populations despite clear genetic isolation by distance. This suggests that elaborate courtship displays can remain remarkably stable even in the face of genetic divergence.

Our results reinforce the notion that many components of courtship displays are conserved over short evolutionary timescales. We observed no additions or deletions of behavioral elements among populations (Fig. 3). Likewise, except for differences in the frequency of “wing-sweep”, the timing of male behaviors (Fig. 4a) and form of female responses (Fig. 4b) were also consistent among populations, and the sequential structure of the display showed no correlation with genetic distance (Fig. 5d). Similarly stable patterns of courtship have been observed in many other taxa, including in consistent expression of the songs of *Drosophila pseudoobscura* populations isolated for >75,000 years (Noor et al. 2000), the leg-waving displays of *Schizocosa crassipes* separated by over 1,000 km (Watts et al. 2019), and in the static calls of many species of hylid tree frogs (Gerhardt 1991). Such patterns of consistency also extend to macroevolutionary scales, such as in the phylogenetic conservation of the tail-straddling walk of *Plethodon* salamanders (Arnold et al. 2017), the bowing display of pigeons (Aves: Columbidae) (Goodwin 1966; Frith 1977), and in the neck displays of phrynosomatid lizards (Wiens 2000). Clearly then, courtship displays can be stable over vast evolutionary periods—but if so, when *does* the process of diversification begin, and how do courtship displays eventually diversify so remarkably among species?

The only detectable divergence among our studied populations was in the frequency of “wing sweep”—with the northern population (Greenpatch beach) performing the behavior less frequently than the two southern populations (Severs beach and Middle beach). Similar quantitative changes have been reported across the animal kingdom such as in the frequency of sigmoid displays of *Poecilia reticulata* (Luyten and Liley 1985), the duration of sine songs in *Drosophila teissieri* (Paillette et al. 1997), and the duration of the long chirp in *Teleogryllus oceanicus* (Simmons et al. 2001). In these examples where courtship does diversify among populations, the changes are most often quantitative modifications of individual elements rather than wholescale rearrangements or deletions of display modules (Hebets et al. 2016; Arnold et al. 2017). Why? Possibly because, over short evolutionary timescales, it is unlikely that whole modules can be reorganized without impacting the functionality of the display (Arnold et al. 2017)—which could greatly reduce mating success and incur a substantial cost to fitness. Nevertheless, there is evidence that with sufficient ecological pressure, saltational jumps in courtship displays can occur among populations over very short timescales. In Hawaiian populations of *T. oceanicus* the entire courtship song was silenced over the span of 20 generations in response to exposure to a novel parasitoid (Zuk et al. 2006; Gallagher et al. 2024).

In the present study, the apparently low rates of courtship divergence may therefore simply be because of limited ecological variation among populations. Ecological variation is expected to be significant across latitudinal gradients—from variation in predation intensity, to the thermal environment and resource availability (Ketterson and Nolan 1976; Barnes 2002; Roslin et al. 2017; Freestone et al. 2021; Lush et al. 2024). Even slight differences in habitat characteristics like wind speed or frequency, sand color, or vegetation types could lead to differences in male display trait optima among populations (as per sensory drive theory; Endler 1992; Cummings and Endler 2018). However, it is possible that beach environments are relatively homogeneous in ecological characteristics such as predation pressure, temperature, and resource availability—particularly over the short latitudinal scales in the present study (<1,000 km). In the context of background variation, which very likely varies among beaches, flies in the genus *Lispe* (Diptera: Muscidae) have shown the capacity to choose consistent display locations (performing their displays preferentially against dark backgrounds of seaweed wrack) which may buffer the signaler-receiver link even in the presence of substantial environmental variation (White et al. 2020)—and it is plausible that *A. ornatipennis* males elicit a similar strategy. The pace of divergence in courtship displays among the studied populations may thus be constrained by both the small scale of ecological differences across beaches and behavioral consistency in the microhabitat choice of displaying males.

However, environmental homogeneity alone cannot explain the lack of courtship divergence among populations—as theory predicts that mutation-order divergence (ie, nonecological diversification) should drive differentiation in sexual traits among populations even in the absence of ecological variation (Mendelson et al. 2014). It is therefore possible that courtship diversification is not stabilized by environmental homogeneity, but instead that there has simply been insufficient time for genetic drift or new mutations to occur in the alleles underpinning male display traits, or, that there are strong pleiotropic constraints (ie, ie, Yeh and True 2014; Ding et al. 2016) or structural constraints (ie, Hebets et al. 2016) on male courtship traits slowing the pace of diversification. Equally, female preference landscapes could underpin the lack of mutation-order divergence. If genetic drift in female preferences is limited, or female preference landscapes are conserved among populations (as has been shown among some populations of *T. oceanicus* for the long chirp; Simmons et al. 2001), then even if new mutations underpinning male courtship traits do arise, they may be rapidly lost from the population.

Regarding female responses, despite clear genetic and geographic differentiation, females across all three populations responded to male display traits in a broadly consistent manner—female walking proportion decreased with increasing male wing sweep and wing flash frequency, while female wing flapping increased with male wing vibrate duration (Table 4). While the form of these relationships was consistent among populations, their strength did vary subtly (Fig. 4b; Table 5). The positive relationship between male wing vibrate proportion and female wing flapping was significantly stronger at Severs Beach than at Greenpatch (Table 5), while Middle Beach, despite its geographic proximity to Severs Beach, did not differ from Greenpatch. The relationship between wing sweep frequency and female walking proportion also showed a marginally significant interaction with population—being slightly stronger in Middle and Severs Beach compared with Greenpatch (Table 5). However, as these differences do not align with the strong patterns of genetic isolation they may only reflect subtle and idiosyncratic variation in the strength of female responsiveness rather than directional divergence in female responses or preference among populations.

Importantly, several caveats qualify these results. Sample sizes (*N* = 45 interacting pairs) and the number of populations examined (*N* = 3) were small, and with 18 discrete behaviors and 41 behavioral states, the risk of Type II error is nontrivial. Greater sample sizes and population replication will be important to corroborate the broad patterns of similarity we detected. Likewise, the 50% occurrence threshold for including behaviors in quantitative analysis, while necessary for replication, may have excluded rare behaviors important for population divergence. Female mating status was also unknown, and copulation was observed only once, meaning most interactions involved unsuccessful courtship; female responses in unreceptive individuals may not accurately reflect preference, and divergence among populations may be more detectable if we had only measured interactions that lead to mating. Finally, our quantitative analyses of courtship timing and female preference used proportional behavioral measures which captures allocation of courtship effort among behaviors rather than the intensity of each behavior, and populations could differ in courtship intensity which may not be detectable in proportional data—ideally interaction times would have been standardized, which would have allowed for comparisons of both courtship allocation and effort, but this was not possible from opportunistic field observations. Importantly, future studies could also benefit from incorporating multimodal courtship traits—including chemical and acoustic signals, which likely complement the visual display in this and related chloropid flies (see Kanmiya 1990; Yatsuk and Shestakov 2022). Likewise, high-speed videography and kinematic analysis could further reveal subtle spatiotemporal features of courtship interactions that diverge among populations.

Overall, our findings contribute to a growing recognition that many components of courtship may be slow to diverge under allopatry. This stability is consistent with uniform selection on choreography among populations, or genetic and structural constraints that limit divergence early in the process of speciation. In many situations therefore, courtship displays may be evolutionarily conserved, diversifying over much longer timescales or only under pronounced ecological variation among populations. Despite this, there are myriad examples of significant diversification in courtship displays among closely related species (Kusmierski et al. 1997; Ligon et al. 2018; Butterworth and Wallman 2021; Girard et al. 2021; Yukilevich 2021). Comparative studies across populations and species using the framework we present here are thus needed across a much wider range of taxa. Such work should involve observations of both sexes and integrate the full suite of courtship components—from differences in qualitative elements, to the timing of behaviors, and display sequence structure. To disentangle the relative contributions of sexual selection and other evolutionary forces to behavioral divergence future studies could also compare rates of divergence across courtship signals, noncourtship behaviors, and broader phenotypic traits—ideally in species where populations span wide geographic ranges with sufficient replication to detect meaningful patterns. Such comparisons would allow an objective assessment of whether courtship traits diverge faster or along different trajectories than traits not subject to sexual selection (eg Tregenza et al. 2007), providing a clearer picture of the role of sexual selection in early divergence among populations.

## Supplementary Material

arag048_Supplementary_Data

## Data Availability

Analyses reported in this article can be reproduced using the data provided by Butterworth et al. (2026). https://doi.org/10.6084/m9.figshare.31669183.
