# Supplementary material for: Courtship choreography is stabilized among genetically isolated populations
Source: Behav Ecol. 2026 Apr 30;37(4):arag048. doi: 10.1093/beheco/arag048 (PMC13180646; doi:10.1093/beheco/arag048)
Supplement: arag048_Supplementary_Data [file arag048_supplementary_data.zip › Supplementary material.docx]

Supplementary material

Courtship choreography is stabilised among genetically isolated populations

Nathan J. Butterworth^1,2^, Thomas E. White^3^, Blake M. Dawson^4^ Jesse Appleton^5^, Clayton McDonald^6^, Angela McGaughran^7^, Gregory Markowsky^6^, Keith M. Bayless^8^

^1^School of Life and Environmental Sciences, Deakin University, Australia

^2^Department of Ecological, Plant and Animal Sciences, La Trobe University, Australia

^3^School of Life and Environmental Sciences, University of Sydney, Australia

^4^Department of Entomology, Michigan State University, United States of America

^5^School of Earth, Atmospheric and Life Sciences, University of Wollongong, Australia

^6^School of Mathematics, Monash University, Australia

^7^Te Aka Mātuatua, School of Science, University of Waikato, New Zealand

^8^Australian National Insect Collection, CSIRO, Australia

**Table S1**. Pairwise Fst values.

|  | **SeversBeach** | **GreenpatchBeach** | **MiddleBeach** | **MerimbulaBeach** | **BearesBeach** | **HaywardsBeach** |
| --- | --- | --- | --- | --- | --- | --- |
| **SeversBeach** | *NA* | *NA* | *NA* | *NA* | *NA* | *NA* |
| **GreenpatchBeach** | 0.2666971 | *NA* | *NA* | *NA* | *NA* | *NA* |
| **MiddleBeach** | 0.0259084 | 0.2565752 | *NA* | *NA* | *NA* | *NA* |
| **MerimbulaBeach** | 0.0139292 | 0.277177 | 0.0133656 | *NA* | *NA* | *NA* |
| **BearesBeach** | 0.1603312 | 0.2834654 | 0.139685 | 0.1592649 | *NA* | *NA* |
| **HaywardsBeach** | 0.1682603 | 0.2810307 | 0.1448951 | 0.1675298 | 0.0207333 | *NA* |
| **WairoBeach** | 0.2429915 | 0.261379 | 0.2199907 | 0.2350984 | 0.1880884 | 0.175054 |


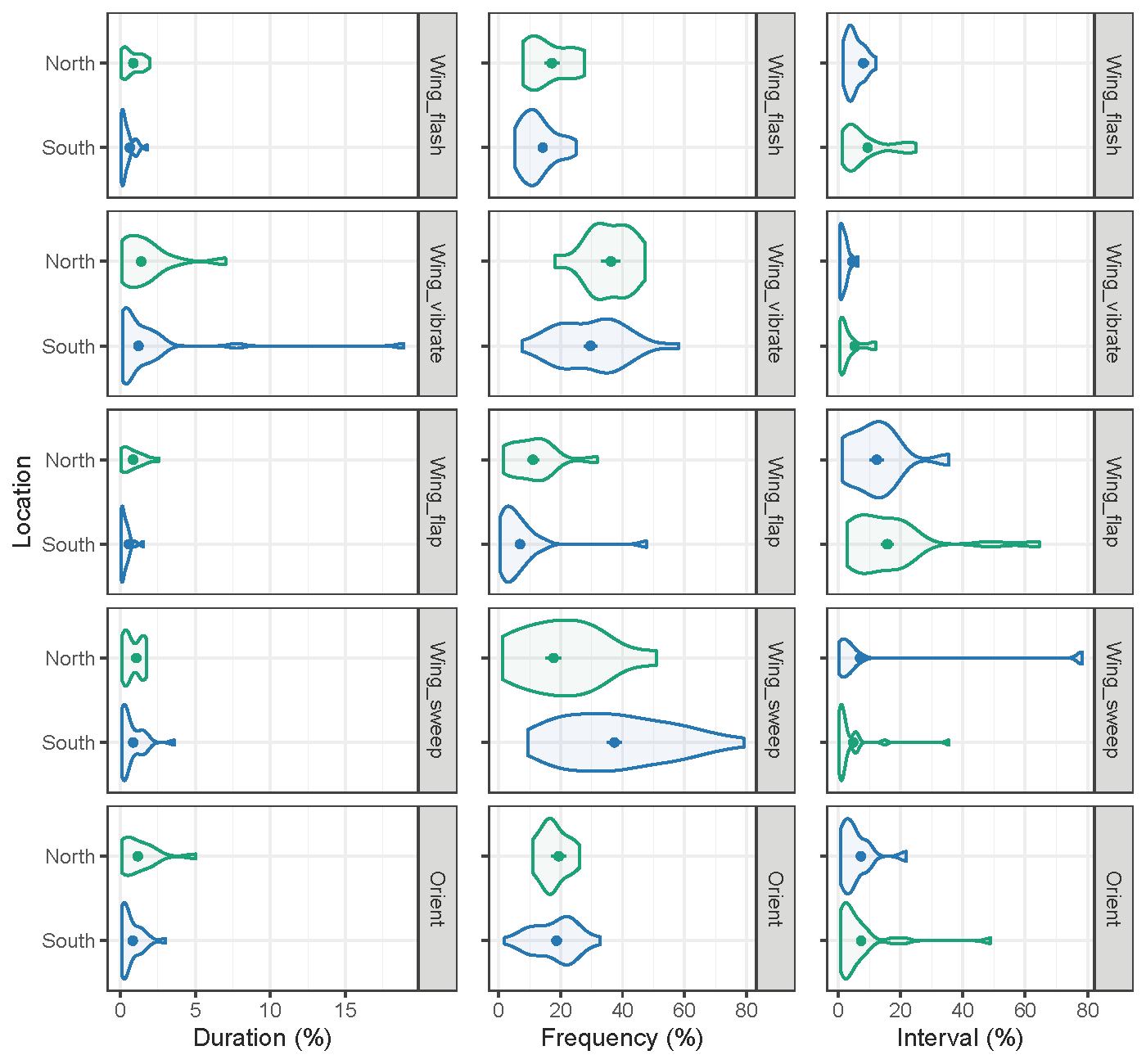
**Figure 1.** Predicted means and standard errors of the bout duration, frequency, and inter-bout interval of key male display components from the beta regression models (Table 2). Location for north is the Greenpatch population, location for south is the combination of Middle + Severs populations.


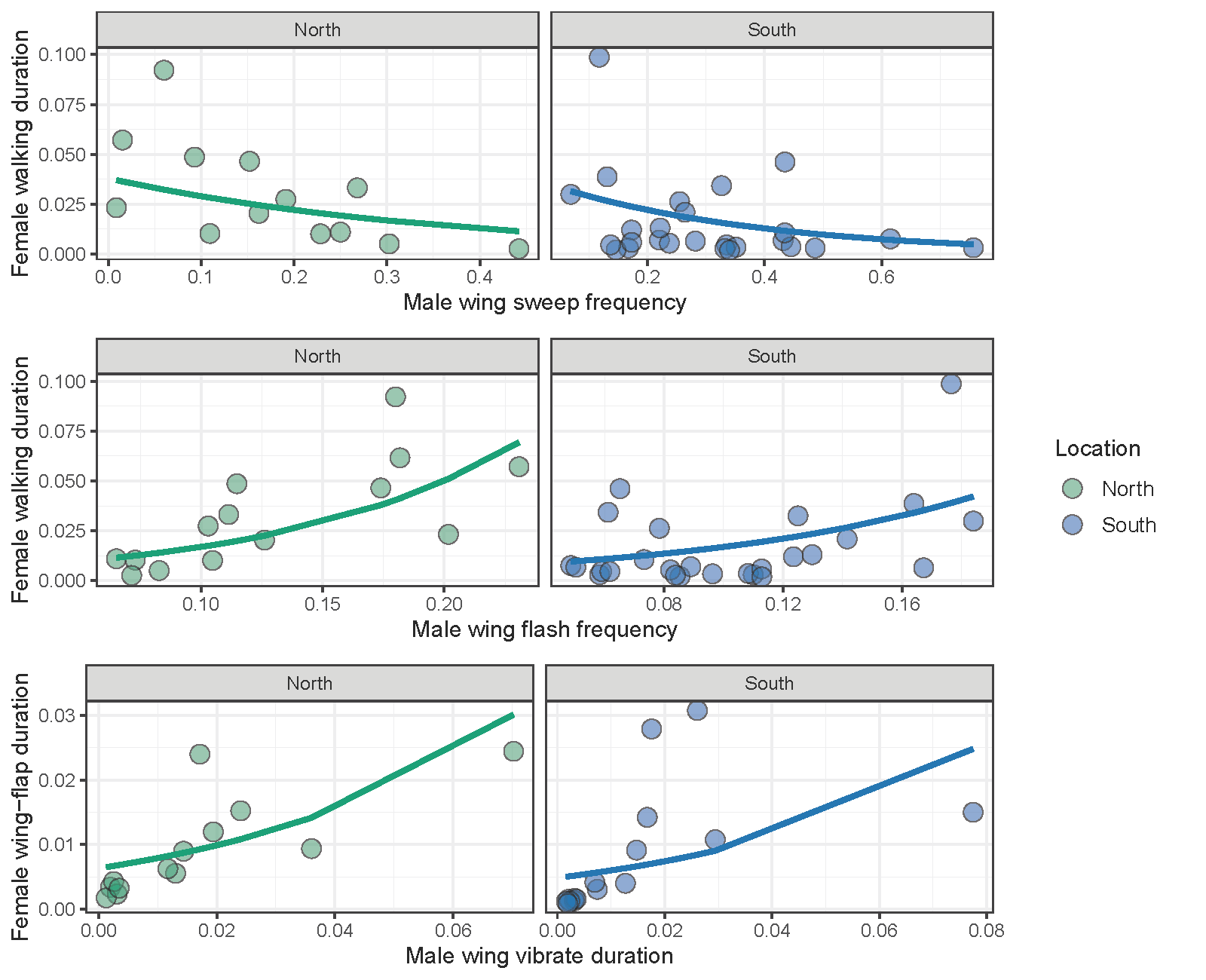


**Figure 2.** Predicted fits of the beta regression models (Table 4) to the correlations between male display behaviours (wing sweep, wing flash, and wing vibrate) and female responses (walking and wing flap). Location for north is the Greenpatch population, location for south is the combination of Middle + Severs populations.

**Table 2.** Result of the analysis of variance (type III) for the effects of location (2 levels) and behaviour (5 levels) on mean proportional duration, frequency, and interval (Supplementary Figure 1). Bold numbers indicate significant values. To account for multiple comparisons, Bonferroni correction was applied (α = 0.01).

|  | **Duration** | | | **Frequency** | | | **Interval** | | |
| --- | --- | --- | --- | --- | --- | --- | --- | --- | --- |
| **Full model** | ***χ2*** | ***df*** | ***p*** | ***χ2*** | ***df*** | ***p*** | ***χ2*** | ***df*** | ***p*** |
| Location | 6.21 | 1 | 0.013 | 0.02 | 1 | 0.875 | 0.12 | 1 | 0.731 |
| Behaviour | 16.39 | 4 | **0.003** | 140.79 | 4 | **<0.001** | 43.81 | 4 | **<0.001** |
| Location*Behaviour | 0.69 | 4 | 0.951 | 35.14 | 4 | **<0.001** | 3.69 | 4 | 0.450 |
| **Wing flash** |  |  |  |  |  |  |  |  |  |
| Location | 2.57 | 1 | 0.109 | 2.78 | 1 | 0.095 | 0.77 | 1 | 0.379 |
| **Wing vibrate** |  |  |  |  |  |  |  |  |  |
| Location | 0.17 | 1 | 0.672 | 4.30 | 1 | 0.038 | 1.00 | 1 | 0.317 |
| **Wing flap** |  |  |  |  |  |  |  |  |  |
| Location | 4.37 | 1 | 0.037 | 4.21 | 1 | 0.039 | 1.11 | 1 | 0.292 |
| **Wing sweep** |  |  |  |  |  |  |  |  |  |
| Location | 1.15 | 1 | 0.283 | 11.40 | 1 | **0.001** | 0.88 | 1 | 0.348 |
| **Orient** |  |  |  |  |  |  |  |  |  |
| Location | 2.38 | 1 | 0.123 | 0.11 | 1 | 0.741 | 0.01 | 1 | 0.963 |

**Table 3.** Results of the analysis of variance (type III) for the effects of male display traits (wing sweep frequency, wing flash frequency, and proportion time spent wing vibrating) on female responses (proportion time spent walking and proportion time spent wing flapping) (Supplementary Figure 2). Bold numbers indicate significant values.

| **Model, parameter** | **ANOVA (Type III)** | | |
| --- | --- | --- | --- |
| **Female walking proportion ~ Male wing sweep frequency * Location** | ***χ2*** | ***df*** | ***p*** |
| Male wing sweep frequency | 8.94 | 1 | **0.003** |
| Location | 5.40 | 1 | **0.020** |
| Male wing sweep frequency* Location | 2.45 | 1 | 0.117 |
| **Female walking proportion ~ Male wing flash frequency * Location** | ***χ2*** | ***df*** | ***p*** |
| Male wing flash frequency | 19.06 | 1 | **<0.001** |
| Location | 0.45 | 1 | 0.502 |
| Male wing flash frequency* Location | 0.01 | 1 | 0.981 |
| **Female wing flap proportion ~ Male wing vibrate proportion * Location** | ***χ2*** | ***df*** | ***p*** |
| Male wing vibrate proportion | 20.87 | 1 | **<0.001** |
| Location | 0.67 | 1 | 0.414 |
| Male wing vibrate proportion* Location | 0.02 | 1 | 0.895 |

**Table 4.** Results of separate beta regression models estimating the effect of male display traits (wing sweep frequency, wing flash frequency, and proportion time spent wing vibrating) on female responses (proportion time spent walking and proportion time spent wing flapping) (Supplementary Figure 2).

| **Model, parameter** | **Estimate** | **SE** | ***z*** | ***P*** | ***R^2^*** |
| --- | --- | --- | --- | --- | --- |
| **Female walking proportion ~ Male wing sweep frequency*Location** |  |  |  |  |  |
| Intercept | -3.30 | 0.21 | -15.22 | **<0.001** | 0.34 |
| Male wing sweep frequency | -2.79 | 0.93 | -2.99 | **0.003** | … |
| South | 0.49 | 0.21 | 2.32 | **0.02** | … |
| Male wing sweep frequency*South | -1.45 | 0.92 | -1.57 | 0.11 | … |
| **Female walking proportion ~ Male wing flash frequency*Location** |  |  |  |  |  |
| Intercept | -4.99 | 0.33 | -15.17 | **<0.001** | 0.37 |
| Male wing flash frequency | 9.94 | 2.28 | 4.37 | **<0.001** | … |
| South | 0.21 | 0.31 | 0.67 | 0.502 | … |
| Male wing flash frequency*South | -0.05 | 2.23 | -0.02 | 0.981 | … |
| **Female wing flap proportion ~ Male wing vibrate proportion*Location** |  |  |  |  |  |
| Intercept | -5.19 | 0.19 | -27.96 | **<0.001** | 0.48 |
| Male wing vibrate duration | 21.95 | 4.81 | 4.57 | **<0.001** | … |
| South | 0.13 | 0.16 | 0.82 | 0.414 | … |
| Male wing vibrate duration*South | 0.62 | 4.71 | 0.13 | 0.895 | … |


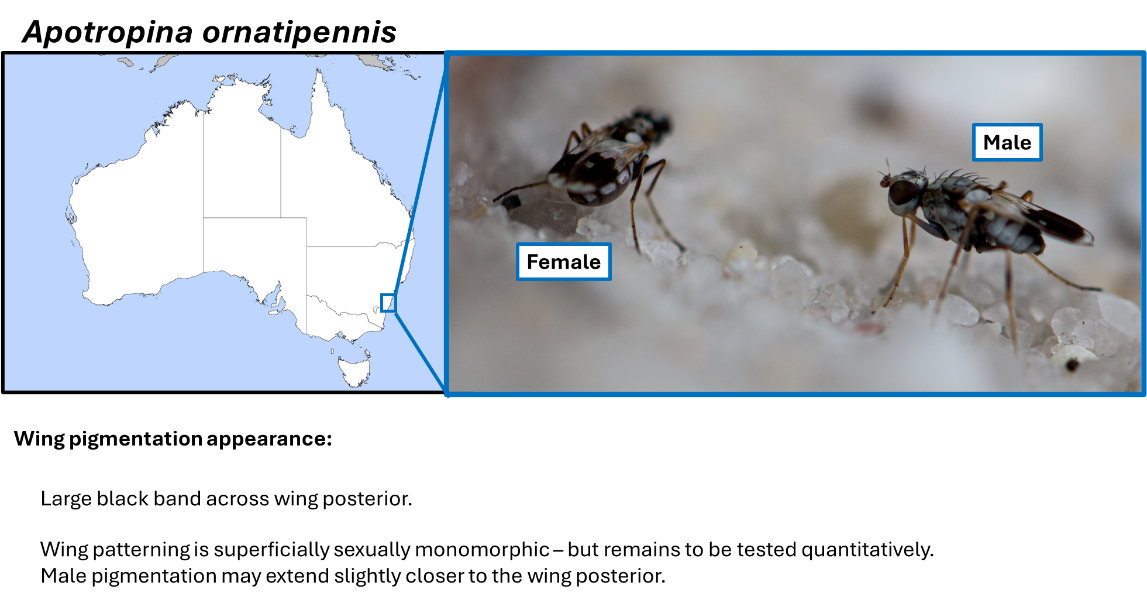

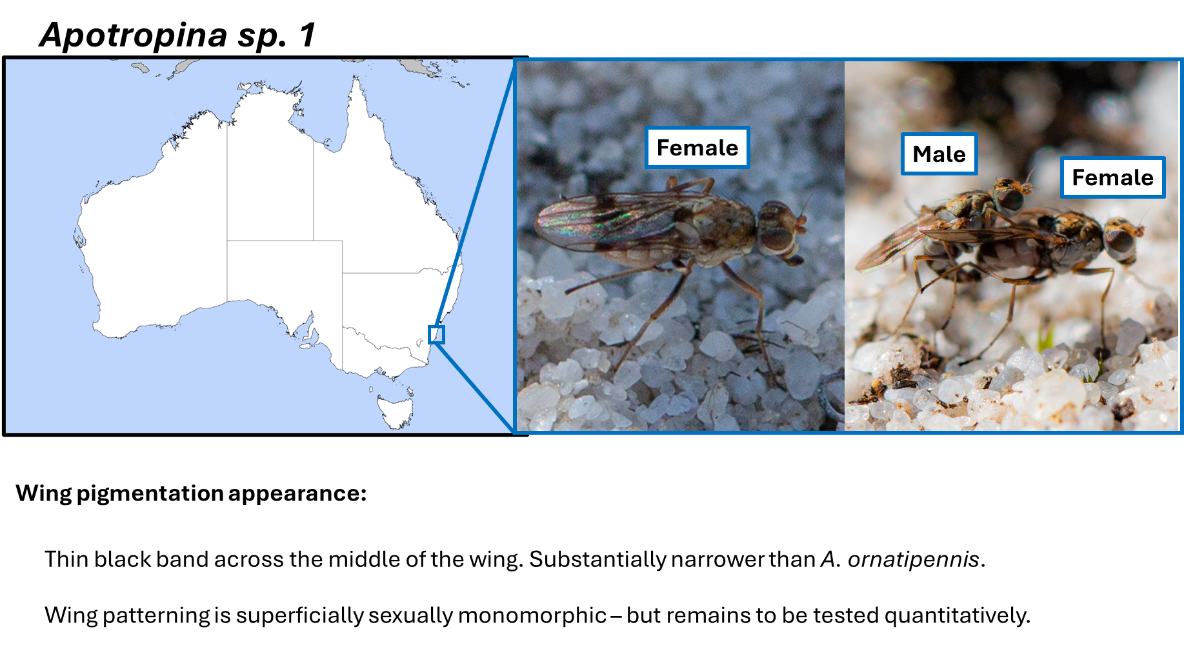

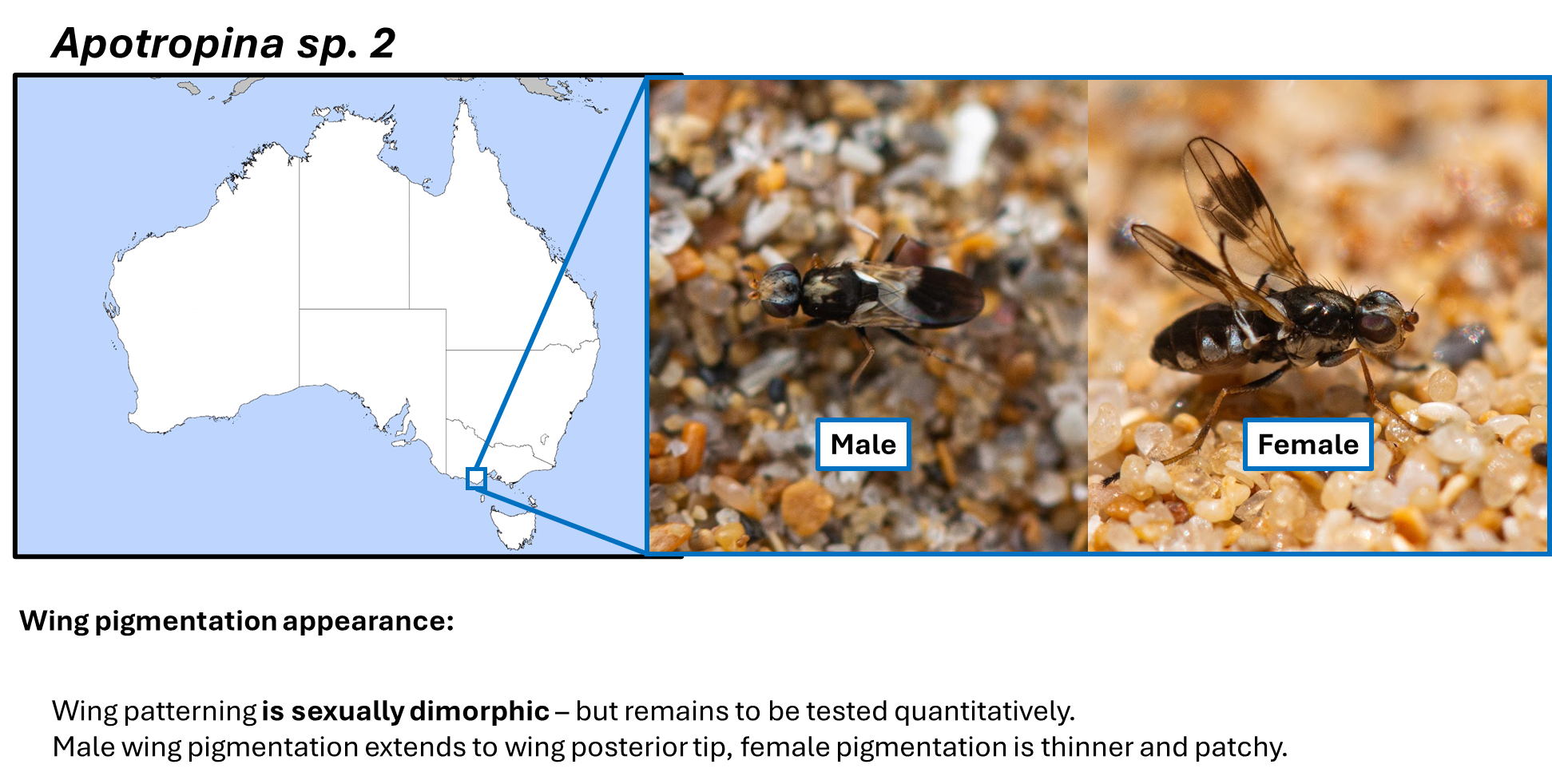


**Figure 3.** Diversity in wing pigmentation patterns across three *Apotropina* species photographed at localities across the Australian coastline. Importantly, designations are tentative and best considered as ‘morphospecies’ as they have not been taxonomically or genetically verified. Top: *Apotropina ornatipennis* which occurs on sand dunes along the coastline of south eastern Australia. Middle: *Apotropina* sp. which was found on a sandy trail near an estuary only ~5km inland from a population of *A. ornatipennis*. Bottom: another coastal species of *Apotropina* which occurs on beaches near the twelve apostles in Victoria. The diversity in wing patterns across species – particularly the sexual dimorphism unique to *Apotropina* sp. 2 in the bottom panel – is consistent with either sexual or ecological selection shaping wing patterning in this genus.
